# Associations of Subjective Social Status with Physical Activity and Body Mass Index across Four Asian Countries

**DOI:** 10.1155/2014/710602

**Published:** 2014-05-28

**Authors:** Leah Frerichs, Terry T.-K. Huang, Duan-Rung Chen

**Affiliations:** ^1^College of Public Health, University of Nebraska Medical Center, Omaha, NE 68198, USA; ^2^Graduate Institute of Health Policy and Management, College of Public Health, National Taiwan University, Taipei 10617, Taiwan

## Abstract

*Objective*. The aims of this study were to (1) assess physical activity and weight status differences and (2) explore the direction and shape of subjective social status (SSS) association with physical activity and weight status within four Asian countries. *Methods*. Cross section data of adult respondents from the nationally representative East Asian Social Survey were used for analyses. Logistic regression stratified by gender was conducted for the first aim, and simple and quadratic logistic regression models were used for the second. *Results*. SSS was significantly associated with odds of weekly or daily physical activity across all countries and genders, except for South Korean and Japanese females. Quadratic models provided significantly better fit for Chinese males (LR (d.f. = 1) = 6.51, *P* value <.05) and females (LR (d.f. = 1) = 7.36, *P* value <.01), South Korean males (LR (d.f. = 1) = 4.40, *P* value <.05), and Taiwanese females (LR (d.f. = 1) = 4.87, *P* value <.05). *Conclusions*. This study provides a comparable cross Asian country measure of moderate-to-vigorous physical activity and new findings that a connection exists between SSS and physical activity. Differences of class distinction help explain the different shaped SSS relationships.

## 1. Introduction

There is considerable empirical evidence that associations exist between socioeconomic status (SES) and weight [1] and SES and physical activity [2]. Across developed countries, relationships of higher physical inactivity rates and increased body mass index (BMI) are found among low SES groups [1, 2]. Historically, the relationships were reversed in less developed countries, but obesity is rapidly rising in low and middle income countries where patterns of sedentary behavior and the burden of obesity shift to lower SES groups as gross national product (GNP) increases [3, 4]. The causal mechanisms that explain the SES-BMI and SES-physical activity relationships are not fully understood. Perception of one's standing within a social hierarchy is postulated to produce motivations, preferences, and opportunities that influence health and health behaviors and may help explain the relationship between SES and health [5, 6].

Many Asian countries are witnessing increases in overweight and obesity [7]. Japan, South Korea, and Taiwan each report combined overweight and obesity prevalence (BMI ≥ 25) in adult populations between 20 and 30% [7–11]. In China, the prevalence is slightly lower, and 19.1% of men and 18.8% of women are overweight or obese; these rates have increased by 414% since the early 1980s [8]. This is particularly a concern due to evidence that the Asian population may have higher susceptibility to chronic health conditions at lower BMIs than other racial groups [12, 13]. The benefits of physical activity on obesity and related health issues (e.g., metabolic syndrome, type 2 diabetes) within Asian populations are well established [14–16], and improving low physical activity levels including domains of leisure and recreation is noted [17].

Social class is central to understanding the factors that naturalize and perpetuate the choices people make in all spheres of their social life that affect their body [18, 19]. SES is commonly measured using income and education, but these metrics may have different implications within and across countries [1] and fail to capture nuances of important social class structures. Subjective social status (SSS), perception of one's social standing in the social milieu, is thought to capture additional pathways and add meaningfulness to the association of SES and health [5, 6, 20]. Research has found that SSS is consistently and often more strongly related to a range of self-reported and objectively measured health conditions including cholesterol levels, hypertension, diabetes, and depression [5, 21–24]. The association of SSS with BMI was not significant in one study [5], but other studies found associations with waist circumference, waist-to-hip ratio, and overweight status [5, 21].

There is considerable economic and social diversity among Asian countries, but little research has looked across countries to better understand variation in health issues and behaviors such as weight and physical activity. Globally, SSS research is limited, and none has considered the potential connections of SSS and physical activity. Furthermore, many SSS studies only assess linear associations, which may fail to uncover more complex social class relationships. The goal of this research was to extend knowledge and explore variation and connections of SSS with weight status and physical activity. Specifically the first aim was to assess weight status and physical activity level differences across four Asian countries. The second aim was to assess if SSS was associated with weight status and physical activity and explore the shape of the relationship within each country.

## 2. Methods

### 2.1. Survey and Sample

Cross-section survey data from the East Asia Social Survey (EASS) 2010 Health Module was used. The EASS is a cross-national project of China, Japan, South Korea, and Taiwan with the intent to research social issues important to East Asian countries. From February 2010 to November 2011, each country fielded the EASS Health Module using multistage stratified probability-proportional-to-size sampling methods to produce nationally representative samples [25]. The survey was conducted using face-to-face interviews, which were either incorporated into a larger survey (China, Japan, and Taiwan) or conducted as an independent study (South Korea). Response rates were 49.48% in Taiwan, 62.1% in Japan, 63.0% in South Korea, and 71.99% in China. The current study included all adult respondents, ages 18–64.

### 2.2. Measures

#### 2.2.1. Demographics

In the present study, sociodemographic variables used included country of current residence, gender, age, marital status, highest level of educational attainment, and occupation classified by manual or nonmanual labor using the Erikson Goldthorpe schema [26].

#### 2.2.2. Subjective Social Status

Respondents rated their SSS using a scale of subjective status [5]. Subjective status scales assess perceptions of social status as they relate to society and are found to have adequate test-retest reliability (*r* = 0.62) [22]. For this survey, a scale numbered 1 through 10, labeling 1 as “Lowest” and 10 as “Highest” was presented and participants were instructed as follows:

*“In our society there are groups which tend to be towards the top and groups which tend to be towards the bottom. Below is a scale that runs from bottom to top. Where would you put yourself on this scale?”*

#### 2.2.3. Moderate-to-Vigorous Physical Activity

Respondents were asked a single-item measure of physical activity, “How often do you do physical activity for at least 20 minutes that makes you sweat or breath heavier than usual?” with response options of “daily,” “several times a week,” “several times a month,” “several times a year or less often,” or “never.” When compared with accelerometry, similar single-item measures are found to reasonably correlate with total physical activity over a one-week period [28, 29], with the highest correlation (*r* = 0.57) with sustained bouts of moderate-to-vigorous physical activity [30]. A dichotomous variable was created to categorize individuals based on expert recommendations for frequency of physical activity (i.e., physically active at least weekly or physically active monthly or less) [31].

#### 2.2.4. Body Mass Index and Weight Status Variables

BMI was calculated from participants' self-reported weight and height (kg/m^2^). Variables to categorize individuals by weight status were created using World Health Organization standard BMI cut-off points (i.e., underweight was defined as a BMI < 18.5, normal weight as a BMI between 18.5 to 24.9, and overweight or obese as a BMI ≥ 25) [27].

### 2.3. Statistical Analysis

Descriptive statistics were used to summarize the sample demographics, physical activity, and weight status by country and gender. Logistic regression models stratified by gender were conducted to determine odds of being (1) physically active at least daily or weekly compared to monthly or less and (2) overweight or obese compared to normal weight between countries adjusting for age, marital status, education level, and manual/nonmanual occupational status.

Simple and quadratic logistic regression models stratified by country and gender were used to examine the association of country-specific SSS quartile on the probability of weekly or daily versus monthly or less physical activity. Multinomial simple and quadratic logistic regression models stratified by country and gender were used to examine the association of country-specific SSS quartile on the probability of underweight versus normal weight and overweight or obese versus normal weight. Likelihood ratio tests were used to determine if the quadratic models provided statistically significant better fit than simple models. The likelihood ratio tests were analyzed separately for binary logistic models of underweight versus normal weight and overweight or obese versus normal weight in order to examine potential differences in the shape of associations. To aid in interpretation, predicted probabilities of daily or weekly physical activity, underweight, and overweight or obese status were calculated from the best fit models. In order to control for potential confounders of SSS, the best fit simple or quadratic regression model for each country and gender was adjusted for age, marital status, education level, and occupation by manual or nonmanual classification.

## 3. Results

### 3.1. Sample Demographics

Table 1 presents a summary of the sample demographics. A total of 8 144 respondents were included in the analysis from China (*n* = 3292), Japan (1725), South Korea (*n* = 1320), and Taiwan (*n* = 1815). China had the highest proportion with lower than high school education (males = 61.6% and females = 66.0%) and who reported occupations classified as manual (males = 51.9% and females = 36.1%). Across countries, the majority of participants were married and few were divorced. The mean SSS was the lowest among Chinese females and the highest among Japanese females. The prevalence of overweight and obesity was comparable to previously reported surveillance data [7, 8]. Using the World Health Organization BMI cut-points [27], overweight prevalence ranges from 10.7% among Japanese females to 34.0% among Taiwanese males.

### 3.2. Physical Activity and BMI across Countries

After adjusting for age, marital status, education level, and manual/nonmanual occupation status, logistic regression analyses revealed significant differences of physical activity and weight status by country (Table 2). Compared to China, Japanese males and females had significantly lower odds of weekly or daily physical activity, OR = 0.59 (95% CI: 0.48, 0.73) and OR = 0.55 (95% CI: 0.44, 0.70), respectively (*P* values < .0001). South Korean and Taiwanese males and females had significantly higher odds of daily or weekly physical activity than Chinese males and females (*P* values < .0001). The odds of overweight or obesity among South Korean males were 1.31 (95% CI: 1.04, 1.65, *P* value < .05) and among Taiwanese males were 2.19 (95% CI: 1.80, 2.67, *P* value < .0001), times higher than Chinese males. The difference between Japanese and Chinese males was not significant. Only Taiwanese females had higher odds of overweight or obesity compared to Chinese females (OR = 1.95, 95% CI: 1.57, 2.43, *P* value < .0001).

### 3.3. Association of SSS Quartile with Physical Activity and BMI

**Figure 1:**
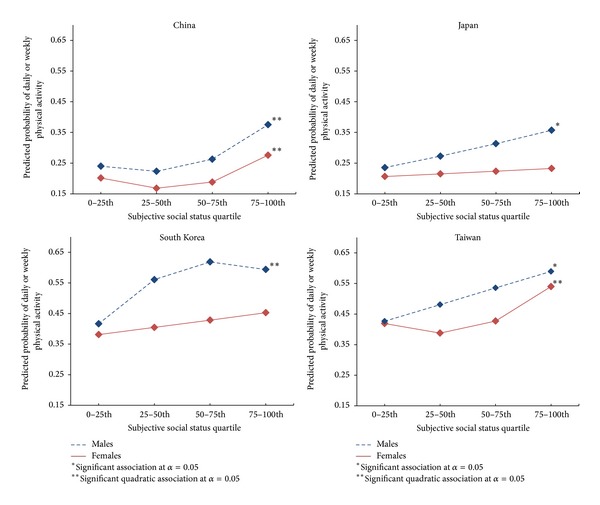
Predicted probabilities of daily or weekly physical activity across SSS quartiles by country and gender. The figure provides a line graph by country and gender of predicted probabilities for daily or weekly physical activity level by SSS quartile from best fit simple or quadratic logistic regression models. The significant associations with only simple SSS quartile terms are indicated with one asterisk, and the significant associations with quadratic SSS quartile terms are indicated by two asterisks. China males and females, South Korean males, and Taiwanese females have significant quadratic relationships, and Japan males and Taiwanese males have significant positive relationships without quadratic terms.

Table 3 provides SSS odds ratios from simple and quadratic logistic regression models for physical activity and weight status outcomes. Increased SSS was significantly associated with increased odds of daily or weekly physical activity for both Japanese males (OR = 1.27, 95% CI: 1.08, 1.49) and Taiwanese males (OR = 1.20, 95% CI: 1.07, 1.36) in simple adjusted models (*P* values < 0.05). Quadratic models provided significantly better fit for Chinese males (LR (d.f. = 1) = 6.51, *P* value < .05) and females (LR (d.f. = 1) = 7.36, *P* value < .01), South Korean males (LR (d.f. = 1) = 4.40, *P* value < .05), and Taiwanese females (LR (d.f. = 1) = 4.87, *P* value < .05). SSS was not associated with daily or weekly physical activity among Japanese or South Korean females.  Figure 1 presents the predicted probabilities for daily or weekly physical activity by SSS quartile for each country and gender.

**Figure 2:**
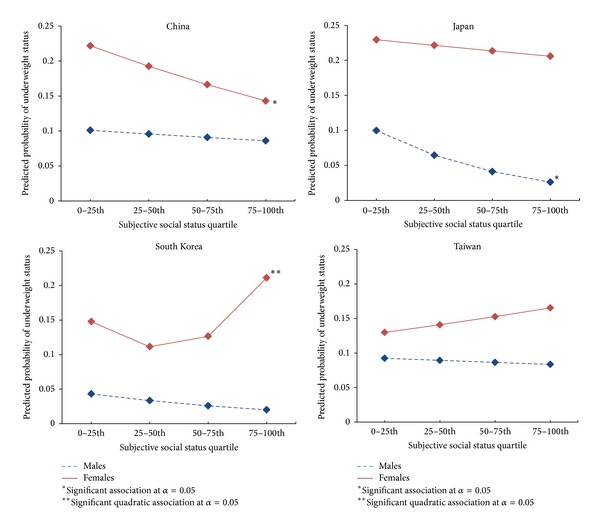
Predicted probabilities of underweight across SSS quartiles by country and gender. The figure provides a line graph by country and gender of predicted probabilities for underweight status by SSS quartile from the best fit simple or quadratic logistic regression models. The significant associations with only simple SSS quartile terms are indicated with one asterisk, and the significant associations with quadratic SSS quartile terms are indicated by two asterisks. Chinese females and Japanese males have a significant inverse relationship between SSS and underweight without quadratic terms. South Korean females have a significant relationship between SSS and underweight status in the quadratic regression model.

**Figure 3:**
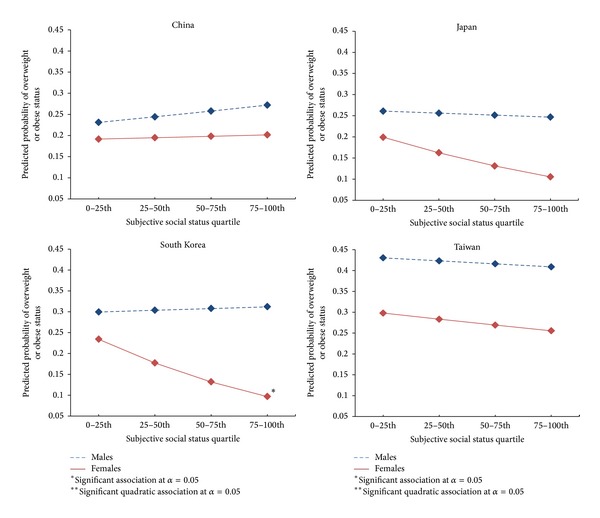
Predicted probabilities of overweight or obesity across SSS quartiles by country and gender. The figure provides a line graph by country and gender of predicted probabilities for overweight or obese status by SSS quartile from the best fit simple logistic regression models. The significant associations with only simple SSS quartile terms are indicated with one asterisk (no significant associations with quadratic SSS quartile terms were found). South Korean females have a significant inverse relationship between SSS and overweight or obesity. No other significant relationships were found.

Increased SSS was significantly associated with lower odds of underweight for Chinese females (OR = 0.84, 95% CI: 0.74, 0.94) and Japanese males (OR = 0.64, 95% CI: 0.44, 0.94) (*P* values < .05). SSS was also significantly associated with underweight among South Korean females in a quadratic model, which provided significantly better fit (LR (d.f. = 1) = 4.06, *P* value < .05). No statistically significant quadratic associations were found in regression analyses for overweight or obese status. Increased SSS was significantly associated with lower odds of overweight and obesity among Japanese (OR = 0.78, 95% CI: 0.62, 0.98, *P* value < .05) and South Korean (OR = 0.71, 95% CI: 0.57, 0.87, *P* value < .001) females in simple models; however, the odds diminished to marginal significance for Japanese females after adjusting for covariates. Figures 2 and 3 present the predicted probabilities for underweight and overweight or obese status, respectively, by SSS quartile for each country and gender.

## 4. Discussion

This study is one of the first to provide a comparable cross Asian country measure of moderate-to-vigorous physical activity. South Korea and Taiwan had higher odds, and Japan lower odds, of weekly or daily physical activity compared to China. In comparison, the International Prevalence Study on Physical Activity (IPPA) indicated that China had the highest activity levels (6.9% low active and 57.7% high active prevalence rates), and Japan and Taiwan had the lowest activity levels (approximately 43.3% and 42.3% low active prevalence rates, resp.) (South Korea was not included in the study) [32]. The definition and measurement of physical activity possibly explain the difference between the studies. In China, a greater proportion of activity is found from work-related and active transportation activities [17, 32], which is captured by the IPPA study measure. This study's measure is more sensitive to shorter bouts of higher intensity physical activity.

Differing values around fitness and exercise across the four countries may also explain the pattern. Japan is noted for positioning itself as a preeminent nation-state that would seek modernization without succumbing to Western domination [33]. The values and obsession with aerobic exercise for fitness and health are largely notions of the American “wellness revolution,” [34] and in the developmental history of sport and body culture, Japan has found ways to subvert Western norms [33].

The prevalence of overweight and obesity in this study was similar to national surveillance data for each country and the between-country patterns found in previous review studies [7, 8]. South Korea and Taiwan had the highest prevalence of overweight or obese status, and China and Japan had the lowest. With the exception of Japan, this pattern follows the positive association between country GNP and weight status [35]. Japan's strong social values and desire for thinness among women compared to other populations [36, 37] and national obesity-related policies across schools [38] and workplaces [39] are possibly exerting strong influence on weight reduction and maintenance.

Results from this study further evidence that a connection exists between perceptions of social class and health, including physical activity, a connection not previously explored. Among males and females, Taiwan and China had positive linear and quadratic associations between SSS and physical activity. Among South Korea males, physical activity showed a quadratic relationship with SSS that formed a u-shaped concave curve. Only a few Asian studies have considered SES and physical activity, which indicated relationships in the same direction. A Taiwanese study found that those with higher education were more active [40], and a Chinese study found that men with higher income had higher odds of regular exercise [41].

Distinctions among low, middle, and high class may help explain the different shaped relationships uncovered between SSS and physical activity. One's perception of social standing may influence decisions to adopt certain behaviors in order to set themselves apart from other SES groups [42, 43]. For example, high status groups may seek to participate in activities that require extensive training or other hidden entry requirements beyond economic costs [43], and sedentary activities such as watching television may be more stigmatized in higher SES households [42]. Taiwan has a relatively robust middle class, which potentially contributes to the linear association found among males [44]. China's middle class has only recently began to grow and remains limited in relative purchasing power and stability [45]. Higher rates of physical activity appear to be emphasized only among Chinese who consider themselves in the highest social class. Additional social or cultural factors likely influence the lower levels of physical activity found among Taiwanese females in the middle SSS quartiles. For example, one study in Taiwan found that females reported lower physical activity self-efficacy and fewer perceived benefits than males [46].

South Korean male's u-shaped concave curve may also be due to a middle class influence. Recently Korean women have become more self-sufficient and “choosy” when selecting a partner, which has led to more Korean men to acquiesce to notions of “kkonminam” in order to set themselves apart from the higher class “businessman” [47]. “Kkonminam” is a pop culture notion of a beauty ideal that reflects a desire to have softer and more feminine features [48] akin to the western notion of “metrosexual” (i.e., one who is especially meticulous about grooming and appearance). Higher levels of activity may be indicative of a desire to participate in more modern sport and recreational activities associated with this type of lifestyle.

This study also provides a cross Asian country analysis of SSS and weight status not previously explored. Increased SSS was associated with decreased probability of underweight among Chinese females and decreased probability of overweight or obesity among South Korean females. Additionally, South Korean females had a significant quadratic association between SSS and underweight status, which reflected that probability of underweight status was slightly lower for the 2nd and 3rd but rose significantly for the highest SSS quartile. Inverse relationships between income or education and obesity risk are fairly consistent across studies among women in Japan [49], Taiwan [9, 50], and South Korea [51, 52]. However, among Chinese women, one study found a positive [53] while another indicated an inverse association [3] between education and obesity. The differences in economic development and social class preferences for body weight may contribute to the respective South Korean and Chinese SSS weight relationships. In more developed countries, such as South Korea, the high SES relationship with lower BMI is especially pronounced among women where thinness is viewed as a sign of beauty, fashion, or prestige [1]. Conversely, undernourishment among low SES populations in China remains a considerable challenge [54].

This study did not find any significant relationships between SSS and overweight or obese status among men, but only one association between SSS and underweight status among Japanese men. Past studies regarding SES and weight associations among Asian men are inconsistent. In Japan and Taiwan, studies have found insignificant relationships by education level [9, 49]. However, another study in Taiwan found an insignificant association of income level, but an inverse association between education and obesity [50]. In South Korea, an inverse relationship was also found between education and income among men [52] but another indicated a significant positive association [51]. Finally, in China, two studies found positive relationships of SES and weight [3, 53].

In this study, the lack of SSS weight status association may also be due to a confounding influence of diet. This study found that Taiwan males and females, Chinese males and females, and South Korean males all had significant associations of SSS and moderate-to-vigorous physical activity. The rise in BMI in developing countries has been tied to a nutrition transition (i.e., increases in energy dense diets) combined with decreases in activity levels from active transportation and occupational activities that result from globalization, urbanization, and economic development [35, 55]. Evidence shows that obesity burden shifts to lower SES groups through this process [3]; however, the combination of physical activity and dietary patterns across social class would enhance understanding of these associations.

This study has several limitations. It is a cross-sectional and associations cannot be assumed as causal links. Further, the physical activity measure was self-reported and of limited precision for measuring specific activities across all domains (e.g., work and transportation). The single-item measure in the current study is most similar to validated physical activity questionnaire items that seek moderate-to-vigorous activities beyond work and active transportation [56]; however, participant's interpretation of the question potentially included types of activity across domains. Thus, interpretations of differences across countries may be problematic due to different patterns of SES status linked to physical activity (e.g., occupational status). Finally, nutrition measures were not available in order to further adjust for potential confounders regarding the associations of SSS and weight. Nevertheless, this study provided a new look at SSS and health not previously explored within a population of increasing need for obesity prevention intervention.

This study provides important findings that can guide future research. The connections of SSS and health compel further exploration of psychosocial factors and pathways. Psychological factors such as stress, negative affect, and coping mediate the relationship between SSS and health [5, 22]. Goodman et al. [21] proposed a model that included such mediated paths between SSS and obesity as well as a path for direct influence of physical activity on obesity; however, this study indicates that a direct association between SSS and physical activity also exists. The complex pathways and feedbacks among SSS, stress, physical activity, and obesity need further consideration and research. Individuals may respond to stress by becoming more sedentary, but evidence exists that exercise can produce biological changes that moderate sensitivity to stress exists [57]. A positive association of stress and obesity [58] and inverse association of physical activity and obesity [59] are also established. This study was unable to explore the potential bidirectional mechanisms and pathways due to its cross-sectional nature, but future studies should research the potential associations in order to identify leverage points for physical activity and obesity interventions.

This study's findings have implications for physical activity intervention development and research. Several of the countries in the current study are implementing physical activity social marketing campaigns [60, 61], a recommended strategy to increase physical activity [62, 63]. In order to develop and target campaign messages, social norms and values must be understood, yet there is little research regarding these in Asia. Only a few studies have qualitatively assessed such values [64, 65] and largely focused on general health values and competing priorities for time without consideration of the influence of perceptions of social class and norms. Leisure time and recreational activity are not the only solution—diet and other forms of physical activity are critical to also be addressed—but social norms and values around this domain are a piece of the complex solutions needed to combat the growing concern of obesity across Asia.

## Figures and Tables

**Table 1:** Sample demographics stratified by country and gender.

	China (*n* = 3292)		Japan (*n* = 1725)		South Korea (*n* = 1320)		Taiwan (*n* = 1815)	
	Male (*n* = 1590)	Female (*n* = 1702)	Male (*n* = 767)	Female (*n* = 958)	Male (*n* = 634)	Female (*n* = 686)	Male (*n* = 900)	Female (*n* = 915)
Age (mean (SD))	43.1 (12.3)	42.3 (12.0)	45.0 (12.6)	44.9 (12.3)	40.1 (12.1)	39.5 (11.5)	40.5 (13.6)	41.5 (13.4)
Education level (% (*n*))								
<High school	61.6 (979)	66.0 (1123)	8.5 (65)	3.8 (36)	11.6 (73)	14.7 (101)	19.4 (175)	29.7 (272)
High school	20.3 (322)	17.9 (304)	44.4 (340)	52.7 (502)	29.4 (186)	36.0 (247)	30.0 (270)	25.8 (236)
College and above	18.2 (289)	16.2 (275)	47.1 (360)	43.5 (414)	59.0 (373)	49.3 (338)	50.6 (455)	44.5 (407)
Marital status (% (*n*))								
Single	14.3 (227)	9.7 (164)	27.4 (210)	17.0 (163)	34.7 (219)	23.2 (159)	39.3 (353)	29.9 (292)
Married	80.5 (1275)	83.3 (1411)	68.8 (528)	75.9 (726)	59.7 (377)	69.0 (472)	54.4 (493)	60.2 (548)
Divorced/widowed	5.1 (81)	7.0 (118)	3.8 (29)	7.1 (68)	5.5 (35)	7.7 (53)	5.9 (53)	10.0 (91)
Occupation (% (*n*))								
Manual	51.9 (825)	36.1 (615)	40.0 (307)	21.8 (209)	27.4 (174)	15.9 (109)	37.0 (333)	23.0 (210)
Nonmanual	24.8 (618)	25.3 (430)	46.0 (353)	44.2 (423)	49.4 (313)	37.8 (259)	39.0 (351)	42.6 (390)
No current work Income	23.3 (370)	38.6 (657)	14.0 (107)	34.0 (326)	23.2 (147)	46.4 (318)	24.0 (216)	34.4 (315)
SSS (mean (SD))	3.9 (1.7)	4.1 (1.7)	5.12 (1.7)	5.3 (1.6)	4.7 (1.7)	4.7 (1.6)	4.8 (1.6)	5.1 (1.5)
Weight status (% (*n*))*								
Underweight (BMI < 18.5)	6.9 (110)	13.2 (225)	4.0 (31)	18.2 (174)	2.2 (14)	13.1 (90)	5.1 (46)	11.9 (109)
Normal weight (BMI = 18.5 to 22.9)	48.3 (768)	50.0 (851)	46.8 (359)	56.6 (542)	41.2 (261)	60.4 (414)	33.9 (305)	48.3 (442)
Overweight (BMI = 23.0 to 27.4)	35.2 (560)	30.4 (518)	38.3 (294)	20.9 (200)	47.2 (299)	21.9 (150)	44.3 (399)	29.5 (270)
Obese (BMI ≥ 27.5)	9.6 (152)	6.4 (108)	10.8 (83)	4.4 (42)	9.5 (60)	4.7 (32)	16.7 (150)	10.3 (94)
Weight status (% (*n*))**								
Underweight (BMI < 18.5)	6.9 (110)	13.2 (225)	4.0 (31)	18.2 (174)	2.2 (14)	13.1 (90)	5.1 (46)	11.9 (109)
Normal weight (BMI = 18.5 to 24.9)	68.5 (1089)	67.5 (1148)	70.7 (542)	69.6 (667)	67.8 (430)	73.3 (503)	55.1 (496)	63.9 (585)
Overweight (BMI = 25.0 to 29.9)	21.4 (340)	17.3 (294)	20.5 (157)	10.7 (102)	26.8 (170)	12.5 (86)	34.0 (306)	19.5 (172)
Obese (BMI ≥ 30)	3.2 (51)	2.1 (35)	4.8 (37)	1.6 (15)	3.2 (20)	1.0 (7)	5.8 (52)	5.4 (49)

*Asia-specific BMI “trigger” cut-off points [27].

**World Health Organization standard BMI cut-off points [27].

**Table 2:** Proportion and odds ratios of physically active and overweight and obese population by country and gender.

	Percent daily or weekly physical activity (95% CL)	Odds ratio^a^ (95% CI)	Percent overweight or obese (95% CL)	Odds ratio^a^ (95% CI)
Males				
China	31.82 (29.52, 34.11)	Ref	24.59 (22.47, 26.71)	Ref
Japan	27.84 (24.67, 31.02)	0.59 (0.48, 0.73)**	25.29 (22.22, 28.37)	0.97 (0.78, 1.22)
South Korea	55.10 (51.21, 58.99)	1.74 (1.41, 2.16)**	29.97 (26.40, 33.53)	1.31 (1.04, 1.65)*
Taiwan	53.73 (50.47, 56.99)	1.70 (1.41, 2.06)**	39.78 (36.58, 42.98)	2.19 (1.80, 2.67)**
Females				
China	25.89 (23.80, 27.99)	Ref	19.33 (17.45, 21.21)	Ref
Japan	22.91 (20.24, 25.57)	0.55 (0.44, 0.70)**	12.21 (10.14, 14.46)	0.77 (0.58, 1.03)
South Korea	41.87 (38.17, 45.57)	1.73 (1.40, 2.13)**	13.56 (11.00, 16.12)	1.05 (0.79, 1.39)
Taiwan	46.44 (43.21, 49.68)	2.17 (1.80, 2.61)**	24.15 (21.38, 26.93)	1.95 (1.57, 2.43)**

^a^Model adjusted for age, education, marital status, and manual/nonmanual labor.

*P value < .05, **P value < .0001.

**Table 3:** Simple and quadratic SSS quartile odds ratios for physical activity models.

	Simple model		Quadratic term model				Adjusted best fit model^a^			
	SSS ouartile OR (95% CI)	*P* value	SSS quartile OR (95% CI)	*P* value	SSS quartile^2^ OR (95% CI)	*P* value	SSS quartile OR (95% CI)	*P* value	SSS quartile^2^ OR (95% CI)	*P* value
Physically active daily or weekly versus monthly or less										
China										
Males	1.28 (1.17, 1.41)*	<.0001	0.58 (0.31, 1.07)	.083	1.17 (1.04, 1.31)*	.010	0.58 (0.31, 1.11)	.099	1.15 (1.01, 1.29)*	.030
Females	1.21 (1.09, 1.34)*	<.001	0.47 (0.23, 0.94)*	.032	1.20 (1.05, 1.36)*	.007	0.45 (0.22, 0.91)*	.026	1.20 (1.05, 1.37)*	.008
Japan										
Males	1.22 (1.05, 1.42)*	.01	1.42 (0.64, 3.14)	.388	0.97 (0.83, 1.13)	.700	1.27 (1.08, 1.49)*	.004	—	—
Females	1.05 (0.89, 1.25)	.566	1.02 (0.42, 2.48)	.971	1.01 (0.85, 1.20)	.939	1.04 (0.80, 1.50)	.713	—	—
South Korea										
Males	1.26 (1.10, 1.45)*	<.001	3.00 (1.32, 6.82)*	.009	0.84 (0.72, 0.99)*	.037	3.10 (1.31, 7.35)*	.010	0.83 (0.70, 0.98)*	.027
Females	1.10 (0.96, 1.27)	.169	1.18 (0.54, 2.55)	.682	0.99 (0.85, 1.15)	.870	1.10 (0.95, 1.28)	.188	—	—
Taiwan										
Males	1.24 (1.11, 1.40)*	<.001	1.24 (0.64, 2.39)	.524	1.00 (0.88, 1.14)	.988	1.20 (1.07, 1.36)*	.003	—	—
Females	1.21 (1.06, 1.37)*	.004	0.57 (0.29, 1.12)	.103	1.16 (1.02, 1.32)*	.027	0.57 (0.28, 1.14)	.109	1.15 (1.01, 1.32)*	.033

Underweight versus normal weight										
China										
Males	0.94 (0.80, 1.11)	.470	0.44 (0.15, 1.30)	.139	1.16 (0.94, 1.43)	.166	0.98 (0.82, 1.16)	.775	—	—
Females	0.84 (0.74, 0.94)*	.002	0.56 (0.26, 1.21)	.137	1.08 (0.93, 1.26)	.295	0.84 (0.74, 0.94)*	.003	—	—
Japan										
Males	0.63 (0.44, 0.90)*	.010	0.92 (0.15, 5.51)	.922	0.92 (0.62, 1.35)	.669	0.64 (0.44, 0.94)*	.023	—	—
Females	0.95 (0.79, 1.15)	.618	0.55 (0.22, 1.39)	.206	1.12 (0.93, 1.34)	.234	0.95 (0.79, 1.15)	.618	—	—
South Korea										
Males	0.77 (0.48, 1.24)	.289	1.94 (0.12, 31.01)	.639	0.83 (0.47, 1.46)	.507	0.75 (0.44, 1.28)	.293	—	—
Females	1.18 (0.96, 1.47)	.124	0.36 (0.11, 1.16)	.087	1.27 (1.01, 1.59)*	.044	0.23 (0.06, 0.83)*	.025	1.38 (1.08, 1.76)*	.010
Taiwan										
Males	0.96 (0.74, 1.25)	.779	1.04 (0.24, 4.59)	.955	0.98 (0.74, 1.32)	.915	0.88 (0.66, 1.16)	.356	—	—
Females	1.10 (0.90, 1.36)	.357	1.07 (0.35, 3.21)	.910	1.01 (0.82, 1.24)	.955	1.12 (0.91, 1.39)	.288	—	—

Overweight or obese versus normal weight										
China										
Males	1.08 (0.97, 1.19)	.153	1.32 (0.69, 2.52)	.410	0.96 (0.85, 1.09)	.539	1.04 (0.94, 1.15)	.482	—	—
Females	1.02 (0.91, 1.14)	.714	1.44 (0.68, 3.05)	.340	0.94 (0.82, 1.08)	.362	1.03 (0.91, 1.15)	.685	—	—
Japan										
Males	0.98 (0.83, 1.14)	.759	0.55 (0.24, 1.25)	.152	1.12 (0.96, 1.32)	.162	0.85 (0.65, 1.23)	.650	—	—
Females	0.78 (0.62, 0.98)*	.034	1.28 (0.40, 4.23)	.663	0.90 (0.71, 1.14)	.387	0.80 (0.63, 1.01)	.058	—	—
South Korea										
Males	1.02 (0.88, 1.18)	.796	0.70 (0.29, 1.69)	.425	1.08 (0.91, 1.28)	.394	1.02 (0.87, 1.19)	.798	—	—
Females	0.71 (0.57, 0.87)*	<.001	0.38 (0.12, 1.16)	.089	1.14 (0.91, 1.44)	.265	0.79 (0.63, 0.98)*	.029	—	—
Taiwan										
Males	0.97 (0.86, 1.09)	.626	1.09 (0.55, 2.15)	.799	0.98 (0.86, 1.12)	.730	0.97 (0.86, 1.10)	.670	—	—
Females	0.93 (0.80, 1.08)	.352	1.86 (0.83, 4.15)	.131	0.87 (0.75, 1.02)	.086	0.98 (0.84, 1.15)	.821	—	—

*P value < .05.

^a^Model adjusted for age, marital status, education level, and occupational classified by manual/nonmanual labor.
